# Chemical Traits and Microbial Population Characterization of ‘Asprinio’ Grape Must, a Local Vine Cultivated in Campania Region (Italy)

**DOI:** 10.3390/foods14122110

**Published:** 2025-06-16

**Authors:** Nicola Landi, Monica Scognamiglio, Lidia Muscariello, Rosangela Marasco, Alessia Palazzo, Ida De Chiara, Sara Ragucci, Paolo V. Pedone, Antonio Fiorentino, Antimo Di Maro

**Affiliations:** 1 Institute of Crystallography, National Research Council, Via Vivaldi 43, 81100 Caserta, Italy; nicola.landi@unicampania.it; 2 Department of Environmental, Biological and Pharmaceutical Sciences and Technologies (DiSTABiF), University of Campania ‘Luigi Vanvitelli’, Via Vivaldi 43, 81100 Caserta, Italy; monica.scognamiglio@unicampania.it (M.S.); lidia.muscariello@unicampania.it (L.M.); rosangela.marasco@unicampania.it (R.M.); alessia.palazzo@studenti.unicampania.it (A.P.); ida.dechiara@unicampania.it (I.D.C.); sara.ragucci@unicampania.it (S.R.); paolovincenzo.pedone@unicampania.it (P.V.P.); antonio.fiorentino@unicampania.it (A.F.)

**Keywords:** Asprinio, free amino acids, metagenomic analysis, nuclear magnetic resonance (NMR), *Vitis vinifera* L., white wine

## Abstract

‘Asprinio’ grape is used to produce a white wine from the Campania region, known as ‘Asprinio’ (DOC since 1993). A plethora of approaches was adopted to characterize the organic compounds (e.g., free amino acids and other metabolites) and microbial population (bacteria and fungi) in ‘Asprinio’ grape must by collecting samples from three different vineyards cultivated with the ‘alberata’ training system. The average free amino acid content of ‘Asprinio’ grape must showed quantitative variations, but no significant statistical differences were found. On average, proline was the most abundant free amino acid (~282 mg/L; 47.9%), followed by arginine (~66 mg/L; 11.5%) and glutamine (~25.2 mg/L; 4.2%). On the other hand, the total polyphenol content (TPC) of ‘Asprinio’ grape must was different, like their antioxidant activity, which increased when the TPC content was higher. Moreover, 1D and 2D NMR spectra highlighted the presence of high sugars amount (in particular glucose) as well as *trans*-caftaric acid, *trans*-coutaric acid, *trans*-fertaric acid, and the *cis*-isomers of these cinnamate esters. Finally, the evaluation of the microbial communities in the ‘Asprinio’ grape must revealed the presence of several representative bacterial phyla—mainly *Bacteroidota*, *Proteobacteria*, and *Actinobacteriota*—as well as various fungal genera, including *Cladosporium*, *Hanseniaspora*, *Aspergillus*, and *Saccharomyces*. Notably, these microorganisms, which contribute to the fermentation process and influence the final wine flavor, have been found in different proportions in the grape musts analyzed. Our results contribute to increasing knowledge of the ‘Asprinio’ grape, an indigenous vine of Southern Italy.

## 1. Introduction

‘Asprinio’ wine (from the Latin term *asper*) is a white wine produced in the Campania region (Southern Italy), which has been recognized as a controlled designation of origin (DOC) product since 1993 [1,2]. This characteristic wine has an intense straw-brilliant yellow color and transmits an intense fresh taste with aromatic notes of citrus, yellow melon, and minerals finish [3]. In recent years, the high acidity (strong freshness) and low alcohol content (~11%) have encouraged ‘Asprinio’ wine consumption, which is considered excellent for pairing with pizza, buffalo mozzarella, seafood, and light starters, becoming an appreciated culinary heritage of the Campania region [3]. In addition, a recent study has highlighted the potential of ‘Asprinio’ wine and grape waste as a model for implementing the bioeconomy, which would benefit local farmers and help preserve biodiversity [4].

‘Asprinio’ wine is produced from the ‘Asprinio’ vine cultivated in Aversa territory, province of Caserta, also known as ‘agro-aversano’. Indeed, the traditional training system of the ‘Asprinio’ grapevine, known as ‘alberata’ or ‘vite maritata’ [5], is typical of ‘agro-aversano’. This training system, developed by Etruscans, is based on living trees (*Populus* spp.) used as poles to tie the ropes on which the vine climbs. This system allows the grapevine to reach considerable heights (~10 m) to receive plenty of sunlight, which is useful for the ripening of the grapes. This peculiar training system has become part of the ‘agro-aversano’ skyline [6].

The origin of the ‘Asprinio’ grapevine is still under debate, although several studies have been carried out [3,7]. Indeed, Spada and co-workers report that it is very similar to ‘Greco di Tufo’ (another grapevine variety cultivated in the Campania region) as reported by Onorati in 1804 [8] and supported by the findings on morphological characteristics [9] and genetic analyses [10,11]. Conversely, other researchers attributed a more ancient origin to this local grapevine, dating it back to the Etruscans [3], justifying the ‘alberata’ training system. In addition, the French origin of this grapevine (19th century) has no historical basis, taking into account some previous historical documents (16th century) [3].

Taken together, all researchers agree that the characteristics of ‘Asprinio’ wine are influenced by the pedo-climatic conditions of ‘agro-aversano’ territory (volcanic origin) and the training system used. Indeed, a recent study on ‘Asprinio’ wine made from grapes cultivated using the ‘alberata’ training system showed chemometric differences with respect to ‘Asprinio’ wine obtained by grapes cultivated using the ‘guyot’ training system like ‘Greco di Tufo’ wine, the latter is another famous white wine of Campania region [12]. The ‘guyot’ training system requires a horizontal structure to support the vines, with taut wires stretched between posts anchored in the ground (height ~2 m) [13].

In this framework, to increase knowledge of the ‘Asprinio’ grape, we decided to perform the chemical characterization of ‘Asprinio’ grape must. This product is typically obtained from the gentle pressing of the grapes, containing water, sugars (170–220 g/L; most of them fructose and glucose), organic acids (9.0–27 g/L; 90% of these tartaric and malic acids), nitrogenous compounds (0.1–1.0 g/L; mostly ammonia, amino acids, polypeptides and proteins), phenolic compounds, and mineral salts [14,15,16]. All these components, on which the fermentative microbial community acts, above all yeasts, give rise to the chemical composition and the character of the finished wine [17,18]. Indeed, glucose and other monosaccharides are the main sources of the carbon necessary for fermentation, and their concentration influences yeast growth [19]. Tartaric and malic acids play a decisive role in the pH of the medium without directly affecting yeast growth [20]. Moreover, ammonia and most amino acids are the main sources of nitrogen for yeast growth, considering that *Saccharomyces* yeasts cannot assimilate inorganic nitrogen (nitrates and nitrites), proteins, or polypeptides [21]. Finally, phenolic compounds are essential for determining wine’s taste and aroma, while mineral salts are necessary for normal yeast metabolism and ion balance [21,22]. In this framework, information about the chemical composition and microbial community in grape must is important for suitable strategies to optimize or improve the fermentation process without compromising the organoleptic characteristics of finished wine [23].

Therefore, the chemical characterization (free amino acid content, total phenolic component, and antioxidant capacity) and nuclear magnetic resonance (NMR) profiles, as well as a metagenomic analysis aimed at evaluating the microbial community of ‘Asprinio’ grape must, were carried out.

## 2. Materials and Methods

### 2.1. Chemicals and Reagents

Folin–Ciocalteu reagent, *nor*-leucine(*nor*-Leu), gallic acid, hydrogen peroxide, and salts were obtained from Sigma-Aldrich Solutions (Merk Life Science, Milan, Italy). Chemicals and solvents for the Kjeldahl method were from Carlo Erba reagents (Milan, Italy), whereas those for automated amino acid analysis were provided by Biochrom (Cambridge, UK). More details for specific reagents or enzymes are reported in the paragraphs below.

### 2.2. Sampling of ‘Asprinio’ Grape Must

‘Asprinio’ grape musts were sampled in September 2023 from three different vineyards (sites) cultivated with ‘alberata’ training system [site 1, named Alberata_A, coordinates: 40°58′10″ N 14°13′52″ E; site 2, Alberata_C, coordinates: 40°57′34″ N 14°13′26″ E; site 3, Alberata_R, coordinates: 40°56′04.4″ N 14°13′33.7″ E] in ‘agro-aversano territory; (Campania region, Southern Italy). ‘Asprinio’ grapes (~5 kg) randomly picked were quickly pressed by using a manual Vevor Fruit Wine Press 12 L (Vevor, Frankfurt, Germany) at 4 °C to avoid fermentation. Subsequently, the grape must was filtered at 4 °C under atmospheric conditions using Miracloth filter paper (Merk Life Science; Milan, Italy).

Finally, clarified grape must samples were centrifuged at 14,000× *g* for 60 min at 4 °C. The supernatant was stored in polypropylene bottles (Falcon, Becton Drive, Franklin Lakes, NJ, USA) at −80 °C until further analysis, while the pellet obtained was freeze-dried and stored at −80 °C for the microbial DNA extraction and high-throughput sequencing (see Section 2.5).

### 2.3. Free Amino Acid Determination

The free amino acid composition of ‘Asprinio’ grape must from Alberata_A, Alberata_C, and Alberata_R was obtained by sampling 1.0 mL (in triplicate) *per* sample. Subsequently, the samples were freeze-dried for amino acid extraction. The freeze-dried powder was subjected first to ethanol precipitation using 80% cold ethanol (1.0 mL) in the presence of *nor*-leucine (200 nmol) as an internal standard, dissolved with a Teflon pestle, and centrifuged at 14,000× *g* for 30 min at 4 °C. The supernatant was lyophilized, treated with 3% sulfosalicylic acid (500 µL) to precipitate any protein fraction still present, and recentrifuged [12]. Aliquots of samples (generally 30 µL) were directly analyzed on a Biochrom-30 amino acid analyzer (Biochrom, Cambridge, UK) equipped with a post-column ninhydrin derivatization system.

### 2.4. Total Phenol Content and Antioxidant Evaluation

*Total phenol content (TPC)*: TPC was determined using the Folin–Ciocalteu procedure on aliquots of 100 µL grape must samples previously diluted (10-fold dilution for each must). The TPC value was expressed as g of gallic acid equivalents (GAEs) *per* L of grape must [12].

*ABTS radical cation scavenging capacity*: ABTS^•+^ solution scavenging capacity of must samples was estimated as previously reported [24]. The results were expressed in terms of TEAC values (mmol Trolox^®^ equivalents *per* L of grape must).

### 2.5. High-Throughput Sequencing

Microbial DNA extraction from each ‘Asprinio’ grape must was performed using Qiagen DNeasy 96 PowerSoil Pro QIAcube HT kit (Qiagen, Milano, Italy), according to the manufacturer’s instructions. The concentration and purity of the extracted nucleic acids were determined by Nanodrop (NanoDrop™ 2000, Thermo Fisher Scientific, Waltham, MA, USA). Next-generation sequencing (NGS) analysis was performed by BMR Genomics S.r.l. service (www.bmr-genomics.it, Padova, Italy; accessed on 2 March 2025).

The microbial diversity was studied by pyrosequencing of the V3-V4 variable region of the 16S rRNA gene, amplified by the universal primer described by Takahashi et al. [25], and the internal transcribed spacer (ITS) ITS2, amplified by ITS3KYO2/ITS4 primers [26]. The amplicon pools, obtained from each ‘Asprinio’ grape must, were purified with the Agencourt AMPure XP kit (Beckman Coulter, Milan, Italy) and re-amplified, including universal primers tailed with Illumina barcode adapters (Nextera XT index kit v2). Amplicons were used for pyrosequencing on the Illumina MiSeq platform (Illumina Italy S.r.l., Milan, Italy) with a 2 × 300 bp paired-end approach. Chimera filtering and OTU clustering were obtained by Qiime 2 using trained sequences (OTUs at 99%) from the Silva ribosomal RNA database (version 138) or UNITE database (version 8.0).

### 2.6. NMR and Partial Purification

NMR samples were prepared as follows: for each sample, 100 µL of phosphate buffer (90 mM; pH 6.0) in D_2_O (Sigma-Aldrich) containing 0.1% *w/v* trimethylsilylpropionic-2,2,3,3-d_4_ acid sodium salt (TMSP; Sigma-Aldrich Solutions, Merk Life Science, Milan, Italy) were added to 900 µL of grape must. Samples were vortexed, and 600 µL were transferred to NMR tubes for subsequent analysis.

NMR spectra of the grape musts were recorded at 25 °C on a Bruker 300 Fourier transform NMR operating at 300.03 MHz for ^1^H and 75.45 MHz for ^13^C. D_2_O was used as an internal lock. No sample rotation was applied. The 1D ^1^H NMR spectra were acquired using a 1D NOESY sequence to suppress the water signal.

Free induction decays (FIDs) were Fourier-transformed, and the resulting spectra were manually phased, baseline-corrected, and calibrated to TMSP at 0.0 ppm using MestReNova software 14.3.1 (Mestrelab Research, S.L.U., Santiago de Compostela, Spain).

In order to obtain fractions enriched in phenolic compounds, 50 mL of each must was chromatographed on Amberlite XAD-4, eluted with water, and then with methanol. The organic fraction was analyzed by NMR.

NMR spectra of the organic fractions were recorded in methanol-d_4_ at 25 °C on a Bruker 500 Fourier transform NMR operating at 500.03 MHz for ^1^H and 125 MHz for ^13^C. Two-dimensional NMR experiments (COSY, HSQC, H2BC, HMBC) were acquired using the standard pulse sequences from the Bruker library.

### 2.7. Statistical Analysis

Analyses were repeated three times for each sample; means and standard deviations (SDs) of the experimental values were reported. Data analysis was carried out with Excel Microsoft 365 (Microsoft Corporation, Redmond, WA, USA). The results were statistically analyzed using a two-way ANOVA test followed by Tukey’s multiple comparisons tests by using the GraphPad Prism 8 software (GraphPad Software Inc., Boston, MA, USA). The significance was accepted for *p* < 0.05 [27].

## 3. Results and Discussion

### 3.1. Free Amino Acid Content in ‘Asprinio’ Grape Must

The grape must obtained from ripe grapes contains approximately 0.1–1.0 g of nitrogenous compounds *per* liter. Free amino acids are the main source of free nitrogen in must, accounting for 25–30% of the total content, contributing to the growth of *Saccharomyces* yeasts [28]. In addition, most of the free amino acids play a role in the formation of several organic compounds (known as aroma compounds) that influence the aroma of the wine [29]. Indeed, several authors affirm that the specific amino acid profile of each grape species influences the aroma of finished wine, although variations due to the specific territory and maturity level can modify the amino acid composition of grape must [29,30]. In particular, some studies report that yeasts use mostly leucine, isoleucine, valine, histidine, glutamine, and proline to produce the aroma compounds found in finished wine [31,32].

Considering this, the free amino acid composition of ‘Asprinio’ grape must from Alberata_A, Alberata_C, and Alberata_R samples was determined using an amino acid analyzer with ninhydrin post-column derivatization.

The type and amount of free amino acids found in each ‘Asprinio’ grape must sample, expressed in mg/L, are reported in Table 1. A comparison of the free amino acid content in Alberata_A, Alberata_C, and Alberata_R must samples showed no significant statistical differences, although there are quantitative differences considering the total free amino acid content of the three samples, which is 646.4, 489.2, and 620.6 mg/L, respectively. Considering the total free amino acids in ‘Asprinio’ grape must samples, proline was the most abundant (~334.5 mg/L (51.7%), ~203.5 mg/L (42.6%), and ~306.9 mg/L (49.5%) in Alberata_A, Alberata_C, and Alberata_R samples, respectively), followed by arginine (8.9%, 15.6%, and 10.1% in Alberata_A, Alberata_C, and Alberata_R samples, respectively) and glutamine (4.6%, 3.4%, and 4.6% in Alberata_A, Alberata_C, and Alberata_R samples, respectively). In particular, the amount of proline and arginine represents the ~60% of total free amino acids in ‘Asprinio’ grape must, in good agreement with the studies published by Petrovic and co-workers, who analyzed different must varieties [33]. However, although proline is the most abundant, it is not a good source of nitrogen for yeasts during fermentation [34]. Indeed, the first nitrogen source is ammonium, followed by arginine, together with glutamic acid, glutamine, aspartic acid, and asparagine, which are the second preferred nitrogen sources for yeasts during alcoholic fermentation [35].

Taking this into account, the data collected showed that, in addition to arginine, ‘Asprinio’ grape must also has good average levels of glutamic acid (28.8 mg/L), glutamine (24.2 mg/L), aspartic acid (13.5 mg/L), and asparagine (6.9 mg/L), which overall account for ~22% of the total free amino acid composition. Moreover, the analysis of ‘Asprinio’ grape must samples revealed the presence of other amino acids not considered a good source of nitrogen for fermentation, such as tryptophan, histidine, glycine, and lysine [35], with average values of 8.2, 9.9, 2.7, and 2.6 mg/L, respectively, which together represent ~4.0% of the total free amino acids.

In addition, considering the average values of the other amino acids retrieved in ‘Asprinio’ grape must samples, the most abundant are GABA (24.7 mg/L), alanine (15.7 mg/L), threonine (14.3 mg/L), serine (13.4), and valine (12.9 mg/L), which represent 2–4% of the total amino acid content retrieved in ‘Asprinio’ grape must samples analyzed, while the remaining ones have an average percentage content below 1.3%.

**Figure 1:**
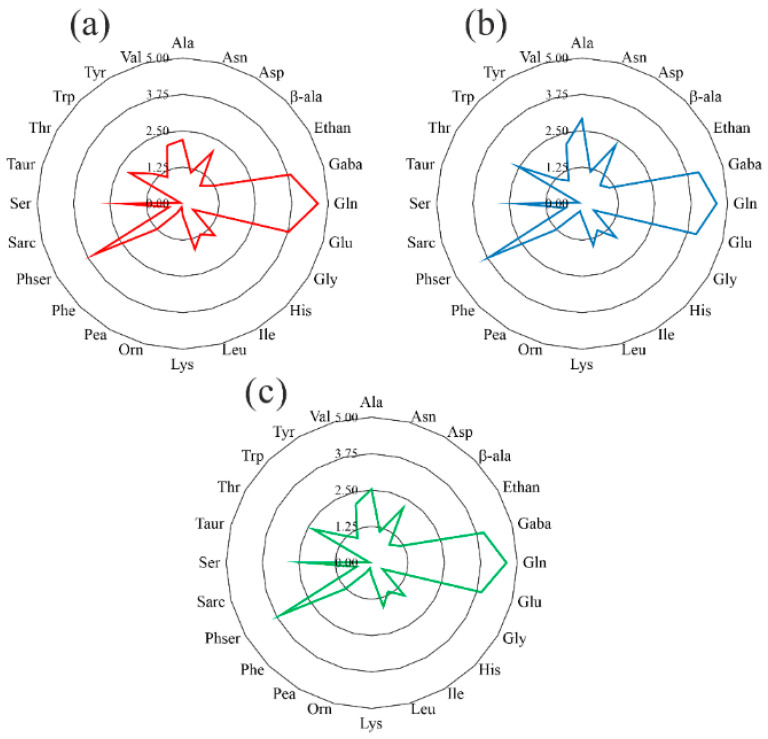
(**a**–**c**) Radar graphs of the average free amino acid profile of ‘Asprinio’ grape must from Alberata_A, Alberata_C, and Alberata_R samples, respectively.

Finally, the amino acid profiles of the three samples analyzed were compared by excluding proline and arginine (Figure 1). The radar graphs show that the three different grape must samples have a similar amino acid profile, providing for a characteristic footprint of the amino acid composition of ‘Asprinio’ grape must obtained from ‘Asprinio’ grapes cultivated according to the agricultural practice of ‘alberata’ training system.

### 3.2. Total Phenol Content and Antioxidant Evaluation of ‘Asprinio’ Grape Must

**Figure 2:**
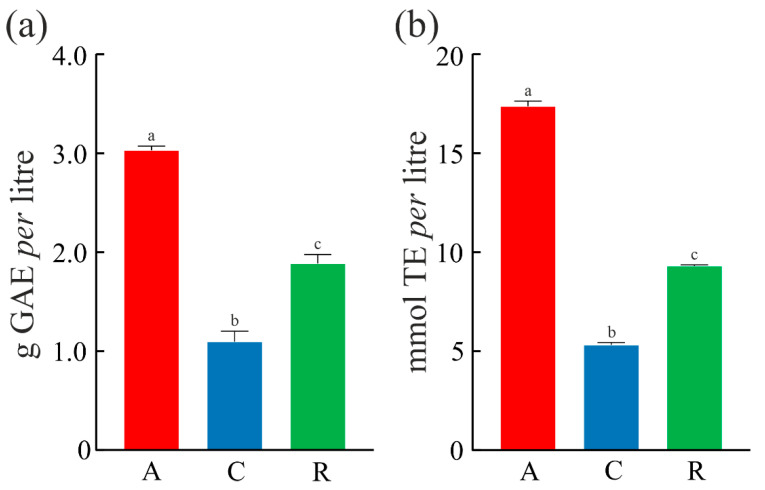
(**a**) Total phenol content (TPC) and (**b**) antioxidant capabilities of Alberata_A, Alberata_C, and Alberata_R ‘Asprinio’ grape musts. In (**a**), TPC expressed as g of gallic acid equivalents (GAEs). In (**b**), ABTS expressed as mmol of Trolox equivalents (TEs) *per* liter. Values are means (±SD) of triplicate analyses (*n* = 3). For each bar, different letters indicate statistical significance according to Tukey’s multiple comparisons test (*p* ≤ 0.05).

Must contains phenolic compounds, most of them endowed with antioxidant activity [16]. Moreover, the levels and composition of phenolic compounds change significantly during fermentation, taking into account their extraction during the increase in ethyl alcohol [36,37]. In this framework, the total phenol content (TPC) of ‘Asprinio’ grape must from Alberata_A, Alberata_C, and Alberata_R was assessed, and the results highlighted a statistical significance between three must samples, as shown in Figure 2a. The data obtained indicate that TPC, expressed as gallic acid equivalent (GAE), was higher in Alberata_A must (3.03 g/L) compared to both ‘Alberata_R’ (1.89 g/L) and Alberata_C musts (1.10 g/L). Although the grapes come from vines grown in the ‘agro-aversano’ territory and with the same training system, differences in local pedo-climatic conditions and solar radiation could explain the variability found [38]. On the other hand, the average TPC (~2.0 g/L) retrieved in ‘Asprinio’ grape must is higher than the average TPC found in ‘Asprinio’ wine produced using grapes collected in the same territory and cultivated with ‘alberata’ training system (~0.6 g/L) [12], confirming a decrease in some of these compounds during the transformation of grape musts into the finished wines [38].

Furthermore, as shown in Figure 2b, the antioxidant activity of ‘Asprinio’ grape must samples evaluated using ABTS highlighted a statistical significance between samples analyzed, displaying that the antioxidant activity, expressed as Trolox equivalent (TE), was higher in Alberata_A grape must (17.37 mmole/L) compared to both ‘Alberata_R’ (9.31 mmole/L) and Alberata_C grape musts (5.31 mmole/L). In light of this, considering both the polyphenol content and antioxidant power of ‘Asprinio’ grape must, a correlation between the TPC and antioxidant activity is evident in all three samples analyzed.

### 3.3. Metabolite Profiling of ‘Asprinio’ Grape Must

**Figure 3:**
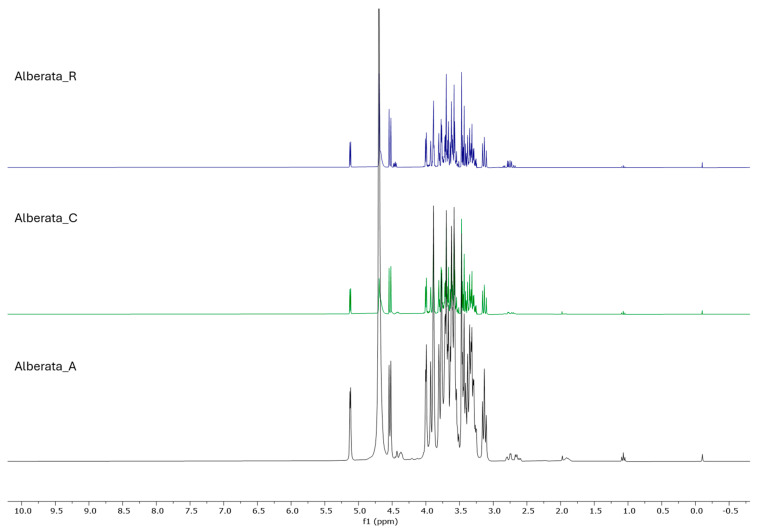
^1^H NMR spectra of Alberata_A, Alberata_C, and Alberata_R ‘Asprinio’ grape must samples. Chemical shifts are reported in ppm with respect to TMS.

The ^1^H NMR profiles of ‘Asprinio’ grape must samples showed the predominant signals belonging to sugars and, in particular, to glucose (Figure 3). Along with glucose, several signals belonging to organic acids, the most abundant one being malic acid, were detected. Furthermore, free amino acids like alanine, leucine, and proline were also detectable. Traces of ethanol were identified thanks to a triplet at δ_H_ 1.07 (-CH_3_), while the other signal belonging to this compound was overlapped with the signals of sugars. NMR signals were also present in the aromatic region, but the identification of the compounds was not possible in these samples due to their low abundance.

**Figure 4:**
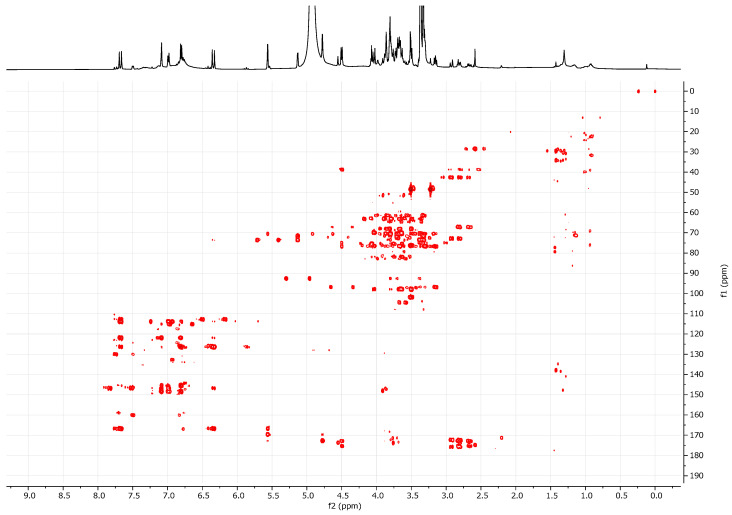
^1^H NMR and HMBC spectra of the partially purified fraction obtained from Alberata_R grape must samples. Data were recorded in methanol-d_4_ at 500 MHz. Chemical shifts are reported in ppm with respect to TMS.

In order to identify the phenolics detected in the grape must samples, an enriched fraction was obtained using Amberlite XAD-4 resin, as described in the methods section. The ^1^H NMR profiles of the ‘Asprinio’ grape musts showed several signals in the aromatic region. The characterization is discussed in the Supplementary Material (paragraph SM1) for the Alberata_R sample. Several 2D NMR experiments were needed for the identification of the main compounds. Relevant information was gained from the HMBC experiment (Figure 4).

Signals of residual aforementioned sugars and organic acids were detected; indeed, as a consequence of their very high concentration, the purification step did not allow us to fully remove these components from the extract.

**Figure 5:**
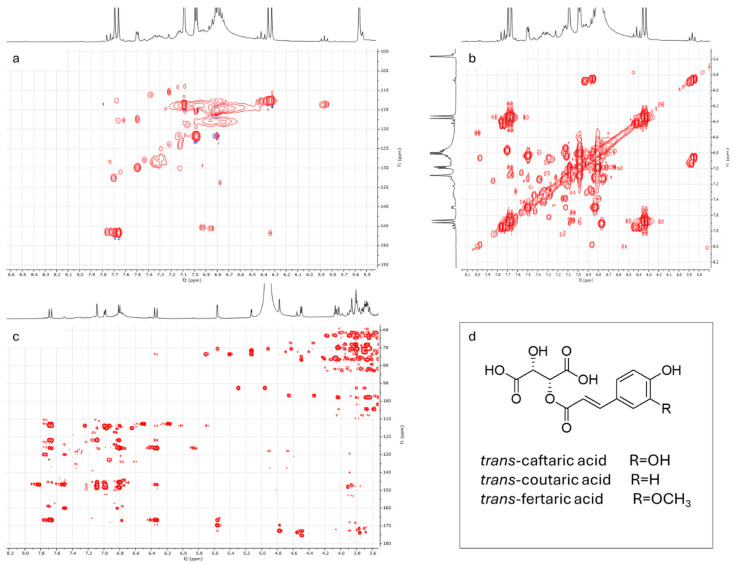
Structural elucidation of the main phenolics in ‘Alberata’ grape must samples. (**a**) Detail of the aromatic region of the HSQC spectrum; the whole spectrum can be found in Figure S2. (**b**) Detail of the aromatic region of the COSY spectrum; the whole spectrum can be found in Figure S3. (**c**) HMBC spectrum: details showing the aromatic region and the relevant correlations that allowed us to identify the main aromatic compounds. (**d**) Trans-cinnamate esters identified in the samples. NMR data were recorded in methanol-d_4_ at 500 MHz. Chemical shifts are reported in ppm with respect to TMS.

However, an extensive 2D NMR study led to the identification of the main phenolic compounds in these samples (Figure 5). A set of signals belonging to an *ortho/para* tri-substituted aromatic ring was detected: two doublets at δ_H_ 7.09 (*J_H_* = 2.1 Hz) and δ_H_ 6.80 (*J_H_* = 8.4 Hz) and a doublet of doublets at δ_H_ 6.99 (*J_H_* = 8.4, 2.1 Hz). Based on 2D NMR data, these signals, along with those at δ_H_ 6.34 and 7.67 (*J_H_* = 16.0 Hz), were attributed to the caffeoyl moiety of *trans*-caftaric acid (Figure 5d). The complete structural elucidation of this compound is discussed in the Supplementary Material section.

Two further sets of signals allowed us to tentatively identify *trans*-coutaric acid and *trans*-fertaric acid (Figure 5d and paragraph SM1). Furthermore, the presence of *cis*-isomers of the cinnamate esters was suggested by the signals detected between 5.80 and 5.90 ppm *(J_H_* = 12.7 Hz) and the observed correlations in 2D NMR experiments (see paragraph SM1).

These compounds were detected in the three ‘Alberata’ samples. Future studies might take into account their quantification. However, it is worth noting that the composition of total hydroxycinnamates has been reported to change progressively during vinification. Indeed, after fermentation begins, there is partial hydrolysis of tartaric acid esters to free hydroxycinnamic acids [39].

Among the esters of hydroxycinnamates with tartaric acid identified in this study, the predominant one is caftaric acid. This compound, along with coutaric and fertaric acids, has been reported from several *Vitis* species [40] and white wines [41]. These chemicals and other hydroxycinnamate derivatives are the major phenols other than flavonoids in grapes and wines and are the predominant ones found in white wines, while in red wines, flavonoids are usually more abundant [42]. Caftaric acid has been shown to have antioxidant and anti-inflammatory properties [43,44]. These bioactivities are also common to other structurally related compounds [45]. It is, therefore, very likely that these compounds contribute to the antioxidant activity of the grape must samples (Figure 2).

### 3.4. Composition of Microbial Community in ‘Asprinio’ Must from Different Alberata

**Figure 6:**
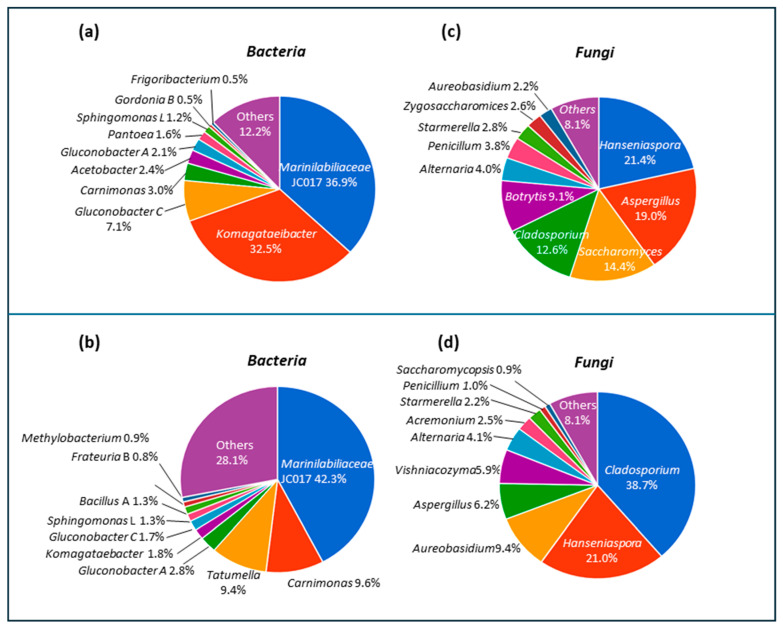
Overview of dominant bacterial and fungal genera in Alberata_C (**a**,**c**) and Alberata_R (**b**,**d**), based on the high-throughput sequencing of 16S rDNA and ITS2 regions.

The microbial component of the three grape musts under investigation was analyzed by using a metagenomic method. For bacterial composition, high throughput 16S rDNA sequencing resulted in 100,605 and 68,222 row reads obtained from Alberata_C and Alberata_R, respectively (Figure 6a,b, Bacteria). After the filtering step, clustering of 69,213 (Alberata_C) and 49,869 (Alberata_R) reads revealed 184 and 165 Amplicon Sequence Variant (ASV), respectively. The ASV genus abundance profile (Figure 6a) shows that Alberata_C sample bacterial communities are constituted by phyla *Bacteroidota*, mainly *Marinilabiliaceae* JC017 (36.9%) and *Proteobacteria*, including *Komagataeibacter* (32.5%) *Gluconobacter* C (7.1%), *Carnimonas* (3%), *Acetobacter* (2.4%), *Gluconobacter* A (2.1%), *Pantoea* (1.6%), *Sphingomonas* L. (1.2%). Less represented the *Actinobacteriota* phylum, with a small percentage of *Gordonia* B (0.5%) and *Frigoribacterium* (0.5%). Bacterial taxa with relative abundance lower than 0.5% are reported as “others” in Figure 6a and include *Firmicutes* (*Enterococcus, Lactococcus, Bacillus, Staphylococcus, Clostridium*) and other *Actinobacteriota* genera (*Arthrobacter, Kocuria, Brevibacterium, Actinomycetospora, Streptomyces*).

Similarly, in Alberata_R, the ASV genus abundance profile shows (Figure 6b, Bacteria) the prevalence of the *Bacteroidota* phylum (*Marinilabiliaceae* JC017, 42.3%), followed by *Proteobacteria*, including *Carnimonas* (9.6%), *Tatumella* (9,4%), *Gluconobacter* A (2.8%), *Komagataeibacter* (1.8%), *Gluconobacter* C (1.7%), *Sphingomonas* L. (1.3%), *Methylobacterium* (0.9%), and *Frateuria* (0.8%). A small percentage of *Firmicutes* was also found, belonging to *Bacillus* A (1.3%) and other genera with relative abundance of less than 0.5% (*Levilactobacillus*, *Lactiplantibacillus*, *Leuconostoc*, *Streptococcus*, *Lactococcus*, *Priestia*, and *Staphylococcus*). *Actinobacteriota* is also very poorly represented, including *Frigobacterium*, *Microbacteriaceae*, *Kinoecoccus*, *Actinomycetospora*, and *Streptomyces*. These genera are commonly found in soil, phyllosphere, and vineyard environments and are well-known for their roles in decomposition, secondary metabolite production, and plant–microbe interactions [46,47]. Although these are also common in wine fermentation and vineyard soils, their low abundance in musts may result from selective pressure exerted by the high sugar content, acidic pH, and anaerobic microenvironments typical of early fermentation, which tend to favor taxa such as *Komagataeibacter*, *Gluconobacter*, and *Marinilabiliaceae JC017*.

Overall, the bacterial communities of the two samples analyzed include genera commonly detected in the wine environment and grape must, such as *Marinilabiliaceae*, *Tatumella*, *Sphingomonas*, *Pantoea*, and *Bacillus* [48,49,50]. The *Tatumella* genus, found only in Alberata C, has been reported to be dominant in grapes in particular climatic conditions [51]. Moreover, the significant presence, in both musts, of the *Carnimonas* genus, abundant in several fermented vegetables and in the gut of some bees, could be associated with insect activity [52,53]. Our data also show that Alberata C has a higher percentage of acetic acid bacteria than Alberata R. Members of this group of bacteria are capable of oxidizing ethanol to acetic acid, causing wine spoilage. Grape musts are often associated with a high abundance of acetic acid bacteria, such as *Gluconobacter*, *Acetobacter*, and *Komagataeibacter*, which can be dominant only in low-sulfite wines [50,54].

For the fungal composition, high-throughput ITS2 sequencing resulted in 197,070 and 138,638 row reads obtained from Alberata_C and Alberata_R, respectively (Figure 6c,d, Fungi). After the filtering step, clustering of 109,642 (Alberata_C) and 90,650 (Alberata_R) reads revealed 147 and 136 amplicon sequence variants (ASVs), respectively. The fungal genera with higher relative abundance for both Alberata_C and Alberata_R were *Cladosporium* (12.6 and 38.7%, respectively), *Hanseniaspora* (21.4 and 21.0%), *Aureobasidium* (2.2 and 9.4%), *Aspergillus* (19.0 and 6.2%), *Alternaria* (4.0 and 4.1%), and *Penicillum* (3.8 and 1.0%). Moreover, *Saccharomyces* (14.4%), *Botrytis* (9.1%), and *Zygosaccharomyces* (2.2%) were only found in the Alberata_C sample (Figure 6c, Fungi), while *Vishniacozyma* (5.9%), *Acremonium* (2.5%), and *Saccharomycopsis* (0.9%) were found in Alberata_R (Figure 6d, Fungi). Finally, other fungal genera, including *Candida*, *Kurtzmaniella*, *Clavispora*, *Pichia*, *Zygoascus*, *Wickerhamiella*, *Stemphylium*, *Pseudopithomyces*, *Keissleriella*, and *Fusarium*, represented less than 1% of the overall abundance in both Alberata_C and Alberata_R samples.

**Figure 7:**
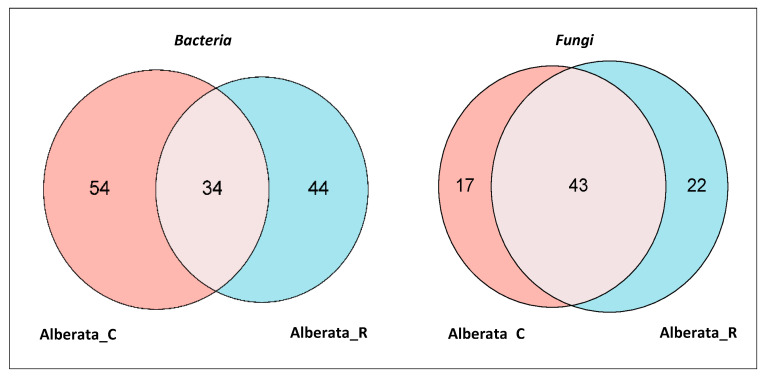
Venn diagrams of the number of shared and unique bacterial and fungal genera in the Alberata_C and Alberata_R.

Overall, analysis of the microbial composition revealed a shared presence of 34 bacterial and 43 fungal genera in both grape musts (Figure 7), which mainly included the most abundant bacterial (*Komagataeibacter*, *Marinilabiliaceae*, JC017, *Carnimonas*, *Gluconobacter*, and *Sphingomonas*) and fungal genera (*Hanseniaspora, Aspergillus*, *Penicillum*, *Alternaria*, *Staramella*, *Penicillum,* and *Aureobasidium*) [49,55]. These results suggest that the microbial communities of the two Alberata are quite similar, although some quantitative differences must be highlighted. For example, *Komagataeibacter* is significantly more represented in Alberata_C (36.9%) compared to Alberata_R (1.7%), and, on the contrary, *Carnimonas* and *Tatumella* are significantly more abundant in Alberata_R (9.6 and 9.4%, respectively) compared to Alberata_C (3.0 and 0.2%). A greater number of differences were observed in the fungal community. Indeed, besides *Hanseniaspora* (21% in both Alberata), the most abundant genera are *Aspergillus* (19.0%), *Saccharomyces* (14.4%), *Cladosporium* (12.6%), and *Botrytis* (9.1%) in Alberata_C, and *Cladosporium* (38.7%), *Aureobasidium* (9.4%), *Aspergillus* (6.2%), and *Vishniacozyma* (5.9%) in Alberata_R.

It is well known that the natural yeasts and bacteria included in the grape must contribute to spontaneous fermentation, impacting the overall fermentation process and influencing the final wine characteristics. The presence of *Saccharomyces* (14.4%) in Alberata_C suggests a strong potential for a faster and more efficient start to alcoholic fermentation, although the presence of generally undesirable genera such as *Aspergillus* and *Cladosporium* and of spoilage bacteria (*Komagataeibacter* and *Acetobacter*) requires careful monitoring of the fermentative process [56].

In contrast, the absence of *Saccharomyces* in Alberata_R indicates that fermentation could be initially supported by non-*Saccharomyces* yeasts like *Hanseniaspora* (21.0%), *Aureobasidium* (9.4%), and *Cladosporium* (38.7%). These yeasts contribute to aroma complexity but may delay the dominance of *Saccharomyces* and slow down ethanol production [57]. Furthermore, some non-*Saccharomyces* yeast genera, including *Aureobasidium*, *Alternaria*, and *Hanseniaspora*, found in both alberata, have been proposed as biocontrol agents against *Botrytis* and other undesirable microorganisms development in early winemaking stages [58]. Anyway, the absence of *Saccharomyces* in Alberata R is not surprising. In fact, it may be related to vineyard-specific factors such as microclimate, grape handling, the timing of sampling, or grape health conditions, which can influence yeast population dynamics prior to fermentation [59,60]. Finally, our data show the presence of *Saccharomycopsis* genus in grape must from Alberata R. Although this yeast does not play a primary role in wine fermentation like *Saccharomyces*, it can be found on grapes and in grape must, where it contributes to enhancing the aroma and complexity of wine [61]. Understanding these complex microbial communities may provide insights into management practices of winemaking to improve individual wine properties.

Unfortunately, we could not obtain valid NGS data from the Alberata_A sample due to the very low amplification of the extracted DNA. This could be due to a technical problem linked to DNA extraction. In fact, DNA isolation from environmental samples is often a critical step in microbial community assessment, especially when performed from preserved samples [62,63]. On the other hand, the grape must sample of Alberata_A could effectively be characterized by a low microbial load. It is well known that the microbial community of grape epidermis, and consequently of the must, can be affected by excessive chemical additives or by weather conditions, such as precipitation, solar radiation, and UV intensity to which vines can be exposed [26].

## 4. Conclusions

In this study, we analyzed, for the first time, ‘Asprinio’ grape must obtained from grapes collected from three different vineyards cultivated with the ‘alberata’ training system.

Our results showed quantitative differences in the metabolite profiles of ‘Asprinio’ grape must without statistical differences. Caftaric acid was identified as the main phenolic compound, along with coutaric and fertaric acids. Moreover, quantitative differences are retrieved also in the taxa of the microbial communities. On the other hand, considering that these microorganisms significantly impact the flavor of ‘Asprinio’ DOC wine, metagenomic analyses highlighted that the different must samples shared 25.7% and 52.4% of bacterial and fungal genera, respectively.

In this framework, this study provides, for the first time, knowledge of the characteristics of ‘Asprinio’ grape must, contributing to highlighting the quality of ‘Asprinio’ wine through the evaluation of the main metabolites already present in the grape must before the fermentation process begins. These metabolites, used as precursors by microorganisms and transformed during fermentation, likely exert a significant influence by modulating the grape must microbial community. In addition, the reported results emphasize the potential of chemometric and ‘omics’ approaches in providing a thorough understanding of complex matrices, such as grape must.

Further research will be carried out to characterize the microbial populations involved in the fermentation process by comparing the grape must obtained from grapes cultivated with the ‘alberata’ training system (traditionally used for ‘Asprinio’ DOC wine) with respect to other training systems (e.g. ‘guyot’).

## Figures and Tables

**Table 1:** Free amino acid composition of ‘Asprinio’ grape must samples from Alberata_A, Alberata_C, and Alberata_R. Values are means (±SD) and are expressed as mg *per* liter of grape must.

Amino Acid ^a^	Alberta_A	Alberata_C	Alberata_R
**Ala**	14.13 ± 1.63 a	18.10 ± 4.97 a	15.72 ± 2.24 a
**Arg**	57.42 ± 4.10 a	76.57 ± 7.36 a	62.78 ± 7.59 a
**Asn**	7.09 ± 1.39 a	5.54 ± 2.48 a	6.90 ± 0.27 a
**Asp**	13.21 ± 1.12 a	12.68 ± 3.33 a	13.46 ± 0.36 a
β-ala	5.65 ± 0.84 a	4.61 ± 1.33 a	5.41 ± 0.21 a
Ethan	7.87 ± 1.04 a	4.76 ± 1.40 a	7.16 ± 1.01 a
GABA	24.81 ± 2.19 a	22.91 ± 4.44 a	24.73 ± 0.11 a
**Gln**	30.04 ± 6.56 a	16.82 ± 18.06 a	28.76 ± 1.80 a
**Glu**	24.34 ± 2.68 a	21.21 ± 6.46 a	24.23 ± 0.16 a
**Gly**	2.72 ± 0.40 a	2.46 ± 0.96 a	2.75 ± 0.05 a
**His**	10.02 ± 0.59 a	8.07 ± 2.53 a	9.87 ± 0.20 a
**Ile**	7.90 ± 0.94 a	4.43 ± 1.39 a	7.11 ± 1.12 a
**Leu**	10.48 ± 1.39 a	6.66 ± 2.18 a	9.67 ± 1.15 a
**Lys**	2.50 ± 0.22 a	2.50 ± 0.75 a	2.60 ± 0.13 a
Orn	0.79 ± 0.01 a	2.06 ± 0.56 a	1.21 ± 0.59 a
Pea	2.49 ± 0.16 a	2.21 ± 0.70 a	2.52 ± 0.04 a
**Phe**	8.04 ± 1.33 a	6.47 ± 2.48 a	7.58 ± 0.27 a
Phser	22.46 ± 1.63 a	20.73 ± 2.68 a	22.21 ± 0.35 a
**Pro**	334.55 ± 29.90 a	203.49 ± 58.65 a	306.87 ± 39.15 a
Sarc	3.14 ± 0.02 a	3.21 ± 0.93 a	3.32 ± 0.25 a
**Ser**	13.45 ± 1.28 a	11.69 ± 3.52 a	13.41 ± 0.06 a
Taur	0.50 ± 0.04 a	0.40 ± 0.41 a	0.56 ± 0.08 a
**Thr**	13.53 ± 1.40 a	14.39 ± 4.5 a	14.30 ± 1.09 a
**Trp**	9.32 ± 1.27 a	3.62 ± 2.86 a	8.17 ± 1.62 a
**Tyr**	6.74 ± 0.89 a	3.82 ± 1.34 a	6.09 ± 0.92 a
**Val**	13.33 ± 1.19 a	9.79 ± 3.84 a	12.91 ± 0.59 a
Total	646.4	489.2	620.6

^a^ Free and protein amino acids. Three-letter codes have been used. Protein amino acids are highlighted in bold. In each row, different letters indicate statistically significant differences according to Tukey’s multiple comparisons test (*p* ≤ 0.05).

## Data Availability

The original contributions presented in this study are included in the article/Supplementary Material; further inquiries can be directed to the corresponding author.

## Supplementary Materials

The following supporting information can be downloaded at https://www.mdpi.com/article/10.3390/foods14122110/s1: Figure S1. HSQC spectrum of the partially purified fraction obtained from Alberata_R grape must samples. Data were recorded in methanol-d4 at 500 MHz. Figure S2. COSY spectrum of the partially purified fraction obtained from Alberata_R grape must samples. Data were recorded in methanol-d4 at 500 MHz. Figure S3. H2BC spectrum of the partially purified fraction obtained from Alberata_R grape must samples. Data were recorded in methanol-d4 at 500 MHz.
