# Supplementary material for: Chemical Traits and Microbial Population Characterization of ‘Asprinio’ Grape Must, a Local Vine Cultivated in Campania Region (Italy)
Source: Foods. 2025 Jun 16;14(12):2110. doi: 10.3390/foods14122110 (PMC12191762; doi:10.3390/foods14122110)
Supplement: Supplementary file 1 [file foods-14-02110-s001.zip › foods-3663132-supplementary.pdf]

SUPPLEMENTARY MATERIALS

SM1: Structural elucidation of the phenolic compounds identified in 'Asprinio' grape must samples

In order to identify the main phenolics in the samples, an extensive NMR study was carried out. A set of signals belonging to an *ortho/para* tri-substituted aromatic ring was detected; two doublets, at δ_H 7.09 (J_H=2.1 Hz) and at δ_H 6.80 (J_H=8.4 Hz), and a doublet of doublet at at δ_H 6.99 (J_H=8.4, 2.1 Hz).

The correlations observed in a long-range (Figure 5a) and in a COSY experiment (Figure 5b), confirmed that these signals all belonged to the same aromatic ring. The long-range correlations shown by two doublets with two carbons at δ_C 148.4 and 148.5 (Figure 5c) suggested the presence of two oxygen bearing aromatic carbons, while the two meta-coupled protons showed a correlation with an olefinic carbon at δ_C 146.7. This carbon showed an HSQC correlation (Figure 5a) with a proton at δ_H 7.67 (J_H=16.0 Hz), in turn showing COSY correlation with a further olefinic proton at δ_H 6.34 (J_H=16.0 Hz). The measured coupling constants suggested a *trans* double bond. Both the olefinic protons showed, among others, long-range correlations with a carboxylic carbon at δ_C 166.7. The described observations allowed us to identify a caffeoyl moiety. This moiety was, however, bound to a further structural unit. Indeed, the carboxylic carbon also showed long-range correlation with a proton at δ_H 5.56 (J_H=2.2 Hz), in turn showing HSQC correlation with a carbon at δ_C 73.5. This carbon showed H2BC correlation (Figure S1) with a proton at δ_H 4.77 (J_H=2.2 Hz), bound to a carbon at δ_C 70.5 (which in turn showed H2BC correlation with the previous proton). The two carbinol protons (δ_H 4.77 and 5.56) showed HMBC correlations with two further carboxylic carbons at δ_C 169.6 and 172.7, respectively. This structural unit esterified with the caffeic acid moiety was identified as tartaric acid, allowing us to identify the compound as *trans*-caftaric acid (Figure 5d).

A second couple of *trans*-olefinic protons was observed at δ_H 6.40 (J_H=16.0 Hz) and 7.75 (J_H=16.0 Hz). The latter proton showed several long-range correlations (similar to the correspondent proton of caftaric acid) and, among these, one with a carbon at δ_C 129.8 which was also correlated with a proton at δ_H 7.49 (J_H=8.2 Hz), showing COSY correlation with a proton at δ_H 6.83. The latter was bound to a carbon at δ_C 114.8. The protons at δ_H 7.49 and 6.83 showed long range correlations also with a carbon at δ_C 159.8. This structural unit was identified as a *para*-coumaric acid unit. The latter also showed correlations with the tartaric acid moiety. Therefore, this compound was tentatively identified as *trans*-coutaric acid (Figure 5d). A third set of signals suggested also the presence of *trans*-fertaric acid (Figure 5d). The presence of the ferulic acid moiety of the latter compound was hypothesized based on the long-range correlation of the characteristic methoxy resonance at δ_H 3.86 with the carbon a δ_C 147.8.

Noteworthy, a set of signals suggesting the presence of the *cis*- isomers of cinnamate esters was detected between 5.80 and 5.90 ppm (J_H=12.7 Hz). These olefinic protons showed COSY correlation with the more deshielded protons in the range between 6.80 and 6.95 ppm (Figure 5b).

Figure S1.

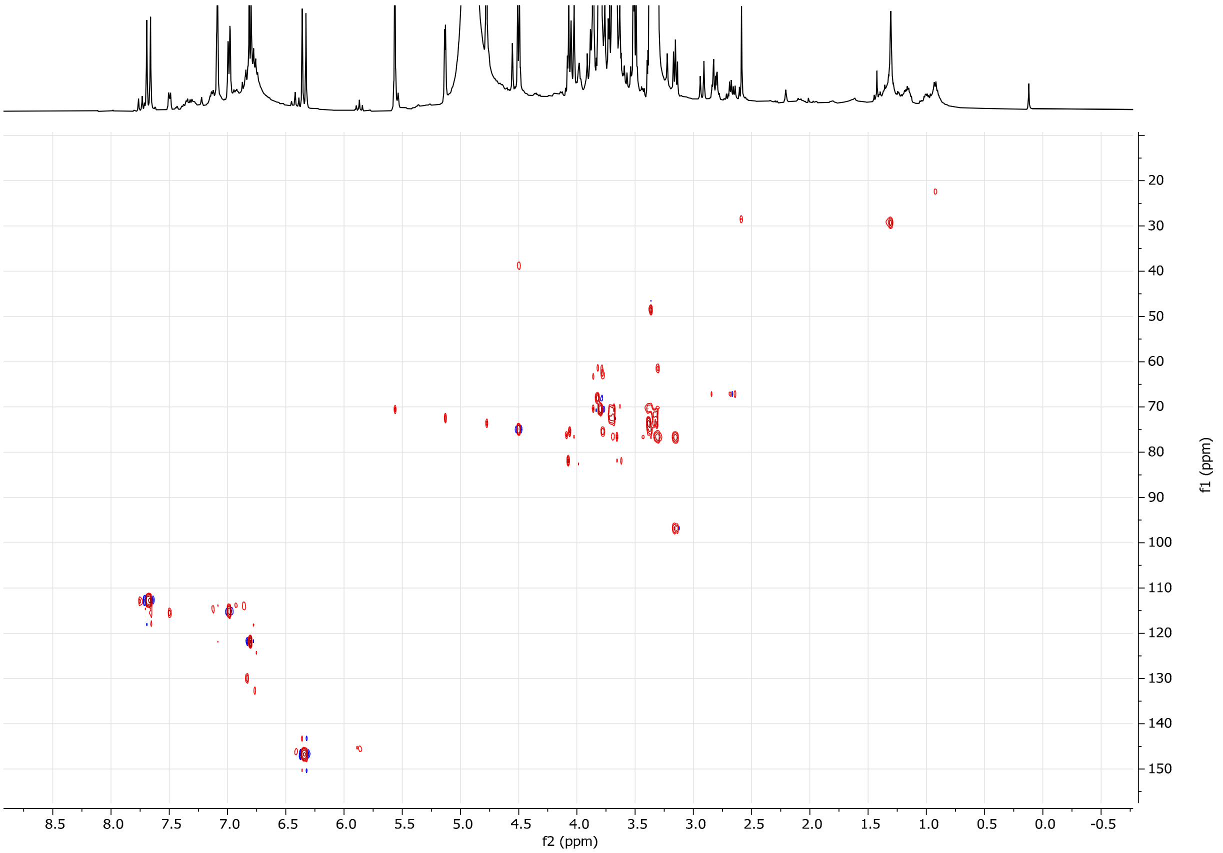

Figure S1. H2BC spectrum of the partially purified fraction obtained from Alberata_R grape must samples. Data were recorded in methanol-d4 at 500 MHz.

Figure S2.

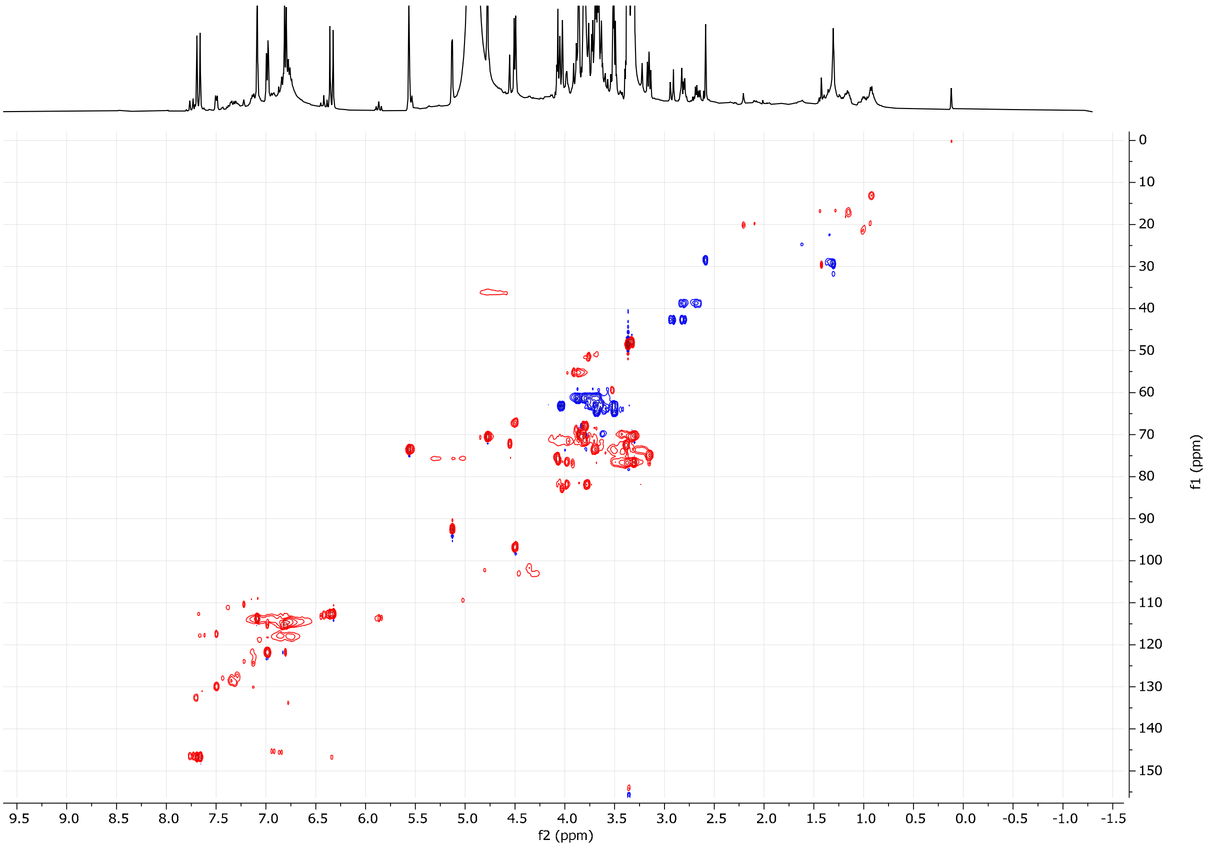

Figure S2. HSQC spectrum of the partially purified fraction obtained from Alberata_R grape must samples. Data were recorded in methanol-d4 at 500 MHz.

Figure S3.

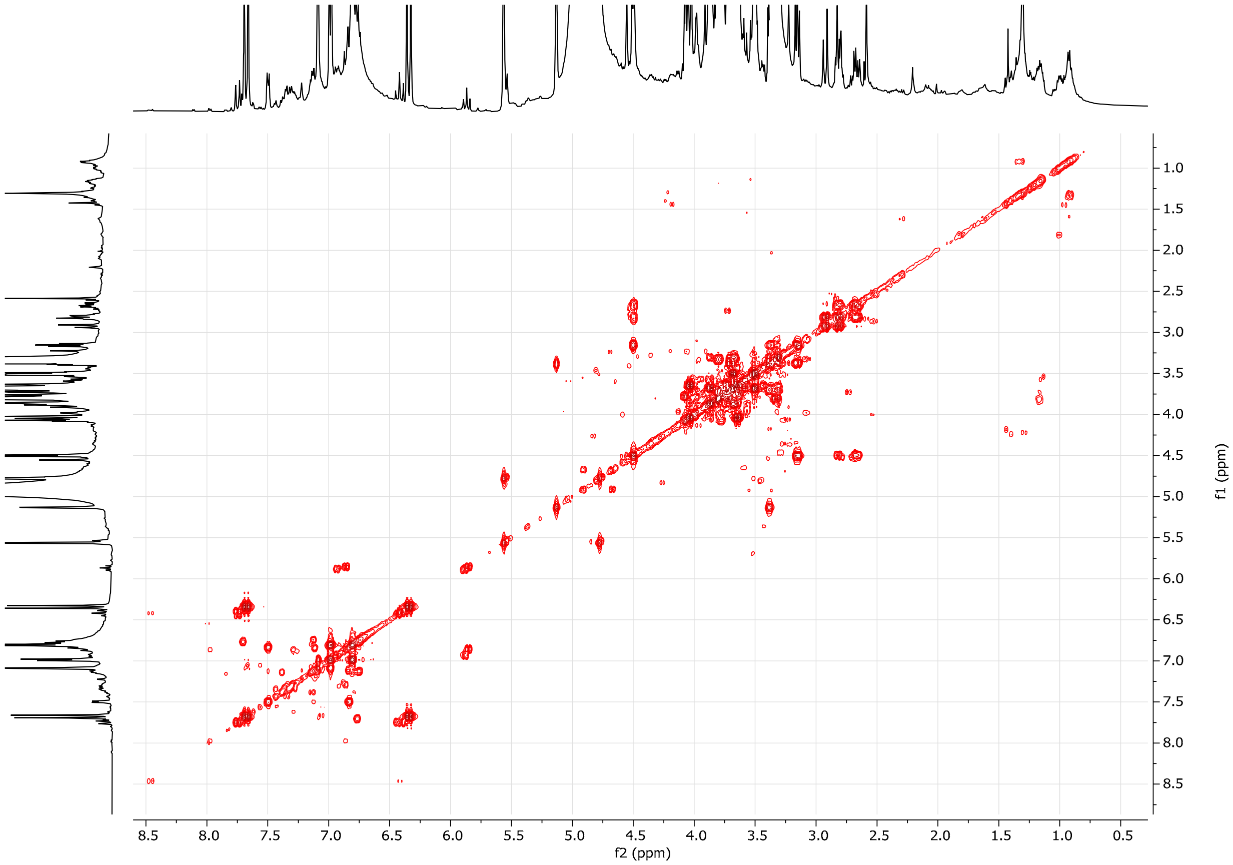

Figure S3. COSY spectrum of the partially purified fraction obtained from Alberata_R grape must samples. Data were recorded in methanol-d4 at 500 MHz.
